# Immune Checkpoint Inhibitor-Induced Ocular Toxicity: A Case of Pembrolizumab-Associated Corneal Ulceration and Evisceration

**DOI:** 10.3390/reports8030154

**Published:** 2025-08-21

**Authors:** Mario Caldarelli, Donatella Brisinda, Giuseppe De Matteis, Francesco De Vito, Gloria Gambini, Rossella Cianci, Giovanni Gambassi

**Affiliations:** 1 General Medicine and Aging Unit, Fondazione Policlinico Universitario A. Gemelli, Istituto di Ricerca e Cura a Carattere Scientifico (IRCCS), 00168 Rome, Italydonatella.brisinda@unicatt.it (D.B.);; 2 Department of Medical and Surgical Sciences, Catholic University of Sacred Heart, 00168 Rome, Italy; 3 Department of Aging, Orthopedic and Rheumatologic Sciences, Catholic University of Sacred Heart, 00168 Rome, Italy; 4 Ophthalmology Unit, Fondazione Policlinico Universitario A. Gemelli, Istituto di Ricerca e Cura a Carattere Scientifico (IRCCS), Catholic University of the Sacred Heart, 00168 Rome, Italy

**Keywords:** immune checkpoint inhibitors (ICIs), immune-related adverse events (irAEs), cancer immunotherapy, visual complications, case report

## Abstract

**Background and Clinical Significance:** Immune checkpoint inhibitors (ICIs) ushered in a new era in cancer treatment, but alongside their efficacy is an adverse event profile that involves the immune system as a whole and may impact several organs. **Case Presentation:** We present the case of a 68-year-old woman with a diagnosis of cervical cancer treated with pembrolizumab who developed progressively steroid-refractory chronic diarrhea and ensuing visual problems. Topical antibiotics failed to heal a corneal ulcer in the left eye, necessitating evisceration. Imaging showed intestinal pneumatosis without ischemia, and there was immediate clinical improvement after initiation of corticosteroid therapy. This clinical picture—steroid-dependent colitis and immune-mediated uveitis associated with secondary bacterial infection—was coded as an immune-related adverse event (irAE) resulting from ICI treatment. Because of the prompt and complete regression of the symptoms upon corticosteroid therapy, this was considered as a criterion for the final diagnosis. **Conclusions**: The case highlights the complexity and potential severity of irAEs that need to be appropriately identified and promptly managed by multidisciplinary teams.

## 1. Introduction and Clinical Significance

Immune checkpoint inhibitors (ICIs) work in the opposite direction of the immunosuppressive pathways, such as CTLA-4 and PD-1/PD-L1; thereby, most effectively, they considerably revitalize immune responses against different types of cancer [1]. The use of ICIs could result in several immune-related side effects [2], in most cases, involving the gastrointestinal tract, skin, endocrine organs, and lungs; however, the involvement of eyes has been reported in rare cases [3].

In addition, it is known that the spectrum of ocular immune-related adverse events (irAEs) includes uveitis, scleritis, and optic neuritis, whereas manifestations like corneal ulceration and perforation are much rarer [4]. Irrespective of the rarity of these diseases, they can sometimes lead to severe consequences, e.g., irreversible vision loss, if the treatment is not immediate. The rarity and severity of ocular side effects, therefore, require a higher level of awareness amongst clinicians of the different medical specialties.

This article is a case of pembrolizumab-related corneal ulceration and colitis in a woman with recurrent cervical cancer. This case not only imparts the unusual potential of the simultaneous occurrence of severe irAEs of the eye and gastrointestinal tract but also delineates the diagnostic challenges in differentiating immune-mediated injury from infections, pointing to the importance of multidisciplinary management strategies. As ICIs become more prevalent in use, oncologists should be alert to early signs and manage these adverse events promptly to save organ function and prolong the active life of patients.

## 2. Case Presentation

A 68-year-old woman presented to the emergency room of the A. Gemelli University Hospital with persistent watery diarrhea lasting six months, for which she had already been previously hospitalized several times. She has a past medical history of cervical cancer diagnosed four years before, for which she was treated with chemotherapy, radiotherapy, and immunotherapy up to about six months earlier. For the treatment of her recurrent disease, she had received IV pembrolizumab at a dose of 200 mg every three weeks from November 2023 to April 2024 (duration of approximately five months). She had no ocular disease and no past or family history of autoimmune disorders. Approximately five months after initiation of therapy, she developed ocular symptoms in the left eye that started with purulent discharge. She had a peripherally inserted central catheter line positioned eight months earlier. Pembrolizumab therapy was discontinued at the time of hospital admission due to the severity of symptoms.

Since the beginning of immunotherapy with pembrolizumab, the patient experienced up to 20 episodes of diarrheal bowel movements per day. Initially, infused steroids were able to modestly reduce the gastrointestinal symptoms, but these became rapidly refractory. Treatment with rifaximin and probiotics also proved ineffective. Approximately five months after initiating pembrolizumab (from November to April), the patient noticed a purulent secretion in the left eye. Home medications included atenolol 100 mg once daily, low-dose aspirin 100 mg once daily, esomeprazole 40 mg once daily, ramipril/amlodipine 5 mg + 5 mg once daily, fenofibrate/simvastatin 145 mg + 20 mg once daily, and levothyroxine 100 μg once daily.

Laboratory tests revealed the following: hemoglobin, 14.7 g/dL (normal range 12.0–15.0); hematocrit, 41.3% (normal range 36.0–46.0); red blood cells, 4.84 × 10^12^/L (normal range 4.50–5.50); MCV, 85.4 fL; MCH, 30.3 pg; MCHC, 35.5 g/dL; RDW, 16.7% (normal range 11.5–14.5); platelets, 350 × 10^9^/L (normal range 150–450); MPV, 10.0 fL (normal range 6.8–10.0); and total white blood cells, 12.01 × 10^9^/L (normal range 4.00–10.00), with neutrophils 8.64 × 10^9^/L (72.0%), eosinophils 0.05 × 10^9^/L (0.4%), basophils 0.04 × 10^9^/L (0.3%), lymphocytes 2.88 × 10^9^/L (24.0%), and monocytes 0.38 × 10^9^/L (3.2%). The leukocyte count showed neutrophilia in the absence of marked leukocytosis of other origin, consistent with an acute infectious process. No lymphocyte phenotyping was performed.

Blood chemistry tests also documented the following: severe renal impairment (creatinine, 4.9 mg/dL; creatinine clearance, 4 mL/min according to the Cockroft–Gault equation), elevated amylase level (280 U/L; normal range ~30–110 U/L), and increased C-reactive protein (CRP), 71 mg/L (normal <5 mg/dL). Abdominal X-ray showed mild gaseous distension of colonic splenic flexure with air–fluid levels in the upper quadrants. Abdominal ultrasound showed no significant abnormalities. The molecular pathogen panel from the stool was negative. Blood cultures were positive for Staphylococcus haemoliticus (peripherally inserted central catheter line positivity time, 4 h; peripheral line positivity time, 10 h).

Serum complement fractions were not measured during hospitalization.

The patient was prescribed antibiotic treatment with meropenem for 15 days and linezolid for 10 days and switched after 48 h to vancomycin due to bone marrow toxicity and drug-induced hypoglycemia for an additional 7 days thereafter.

**Figure 1:**
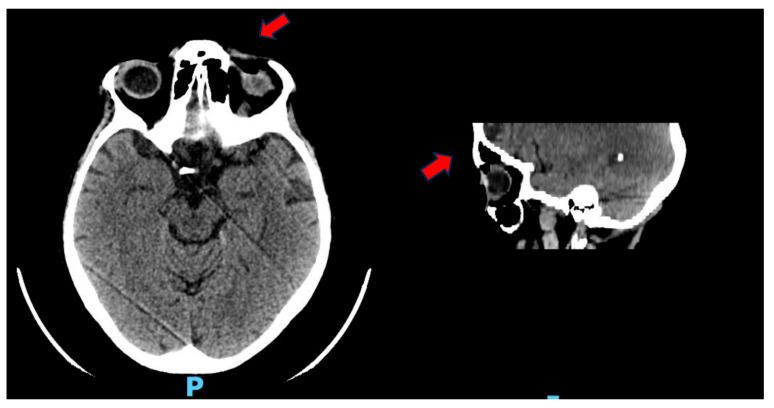
Investigation performed by multilayer spiral technique under baseline and emergency conditions. At the left orbit, minimal edematous suffusion of periorbital and upper eyelid tissue is documented. Concomitant air altitude at the level of the superior conjunctival fornix closely adhered to the surface of the eyeball. Otherwise, no CT evidence of changes of the ipsilateral bulb and retrobulbar soft tissues is documented.

The initial clinical ophthalmological evaluation was followed by an orbital CT, which revealed edematous infiltration of the left periorbital and upper eyelid tissues. An air pocket was also detected at the superior conjunctival fornix, in close contact with the ocular globe (Figure 1). A trial of topical therapy with levofloxacin was initiated, without apparent beneficial effects.

Subsequent ophthalmologic examinations confirmed a corneal ulcer, and the patient underwent lateral tarsorrhaphy and repair of the corneal perforation with a lamellar corneal graft.

**Figure 2:**
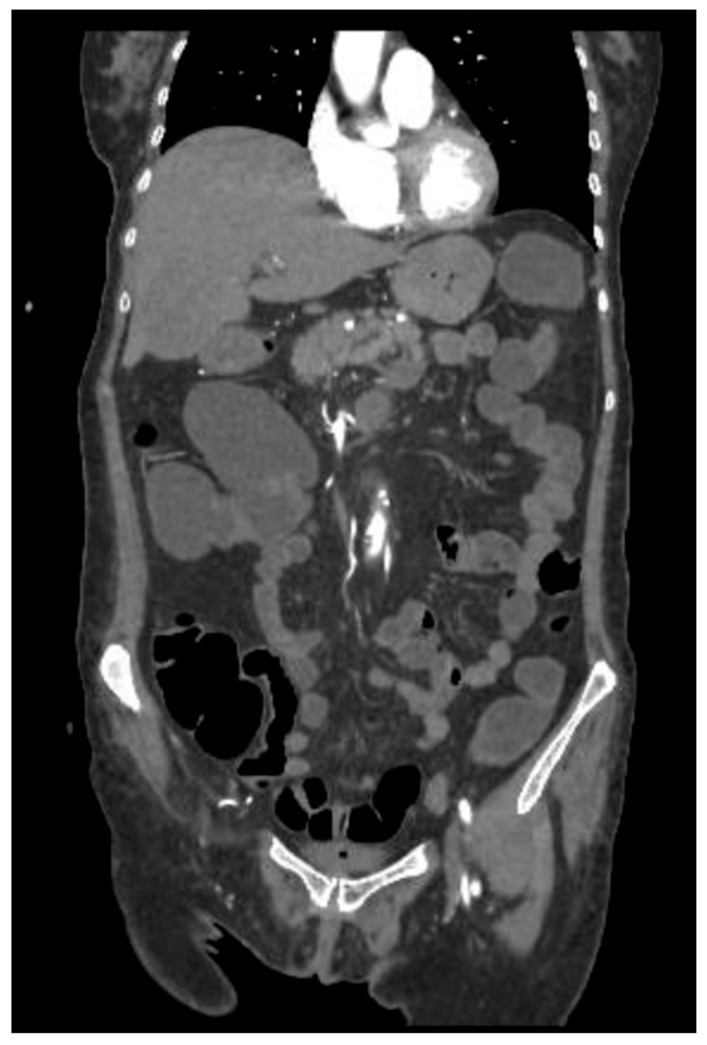
CT scan with contrast medium: hypoperfused appearance of the walls of the transverse colon, with the presence of free air in the context of the walls and around the viscera, as in intestinal pneumatosis.

Three weeks later, however, the patient underwent ocular evisceration due to corneal perforation secondary to corneal melting. HLA typing (including HLA-A29, HLA-B27, and HLA-DR4) was not performed. Corneal scraping and culture were also not carried out prior to surgical intervention. Due to persistent diarrhea and the suspicion of a toxic megacolon, an abdominal CT was performed, revealing intestinal pneumatosis with signs of vascular hypoperfusion in the transverse colon (Figure 2). An exploratory laparoscopy was performed, but there was no evidence of ischemic damage.

Treatment with cholestyramine and rifaximin (400 mg three times daily) was initiated and continued for two weeks with no benefit. The initiation of 0.5 mg/kg methylprednisolone on 30 September 2024, was followed by a gradual normalization of stool consistency. Systemic corticosteroid therapy was started only after proving that source control of the infected corneal ulcer had been established surgically. Clinical and laboratory improvement evidence became evident. During the active infectious phase (early to mid-September), the patient was managed solely on topicals with antibiotics, eyelid hygiene, and protective bandaging without any topical or systemic corticosteroids, so as not to worsen bacterial keratitis. The steroid was purposely delayed to minimize the risk of infection.

Improvements in clinical conditions were noticed within 24 h after initiation of methylprednisolone therapy (0.5 mg per kg), with complete resolution of diarrhea and ocular discomfort. Laboratory parameters were normal at discharge, with the white blood cell count having come down from 12.0 × 10^9^/L to 7.0 × 10^9^/L (reference range, 4.29–10.00 × 10^9^/L) and C-reactive protein from 71 mg/dL to 8 mg/dL. No further abdominal imaging was done during hospitalization, and no other laboratory or ophthalmologic follow-up data were available as the patient was lost to follow-up after discharge.

The unresolving diarrhea during treatment with immune checkpoint inhibitors, with edematous thickening of ascending colon wall and associated mucositis (“sick-sickness-like” syndrome), associated to uveitis complicated by bacterial superinfection, was tentatively ascribed to an adverse effect of immunotherapy. The diagnosis was substantiated by the rapid response to corticosteroid therapy.

**Figure 3:**
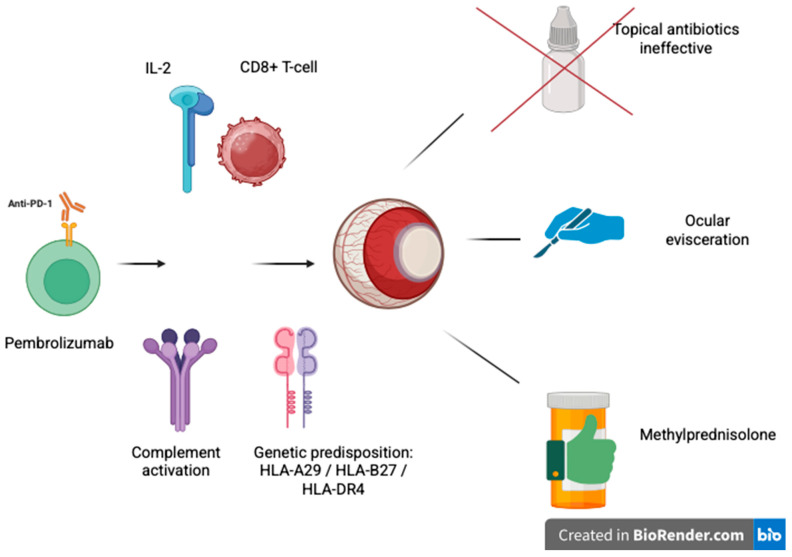
Mechanisms of immune-related ocular toxicity induced by pembrolizumab. Anti-PD-1 therapy enhances CD8^+^ T-cell activation and IL-2 production, potentially triggering complement activation and ocular inflammation in genetically predisposed individuals (e.g., HLA-A29, HLA-B27, and HLA-DR4). These immune responses can lead to ocular damage unresponsive to topical antibiotics, requiring systemic corticosteroids such as methylprednisolone or, in severe cases, ocular evisceration. Created with BioRender.com.

As shown in Figure 3, pembrolizumab may enhance CD8^+^ T-cell activation and IL-2 release, triggering complement activation and ocular inflammation in genetically predisposed individuals.

Alternative explanations for corneal ulceration and colitis were being actively considered and studied. The intestinal pathogen panel did not reveal any abnormalities in the colitis work-up, which helped exclude certain common viral, bacterial, or parasitic etiologies; exploratory laparotomy ruled out possible ischemia of the small bowel despite the presence of pneumatosis on imaging, and inflammatory bowel disease was not reported in the previous history. Orbital CT excluded the possibility of orbital masses or primary structural abnormalities for the ocular lesion, and the previous clinical history had no reported inflammatory or autoimmune diseases. Ocular secretions were negative on microbiological testing. Despite the absence of HLA typing and corneal scraping/culture, the sequence of events, coupled with the improbability of any other systemic inflammatory or infectious causes and the rapid corticosteroid response after surgical source control, suggested an immune-mediated mechanism triggered by pembrolizumab, secondarily complicated by bacterial keratitis.

## 3. Discussion

### 3.1. Immune Checkpoint Inhibitors

Immune checkpoint inhibitors (ICIs) work by blocking mechanisms tumors use to escape immune detection. The immune checkpoints act as negative regulators of the immune system under normal physiological circumstances, allowing self-tolerance and preventing autoimmune damage. However, tumors hijack these pathways to upregulate immune checkpoint proteins on T cells, dampening the host immune response [5].

The best-characterized immune checkpoint pathways include CTLA-4 and PD-1. CTLA-4 is a transmembrane protein present in CD4^+^ and CD8^+^ T cells. The binding of CD80 and CD86 to their ligands on antigen-presenting cells inhibits cytotoxic T-cell function and potentiates immunosuppressive effects in regulatory T cells. CD80 and CD86 are the primary ligands for CTLA-4; their interaction delivers an inhibitory signal that downregulates T-cell activation, thereby maintaining immune homeostasis.

PD-1, another inhibitory receptor, is expressed in T and B cells and NK and dendritic cells. Its main ligand, PD-L1, is found on a wide range of cells, such as epithelial cells, macrophages, and fibroblasts. The interaction of PD-1/PD-L1 inhibits the proliferation and differentiation of T cells [6].

Tumors hijack these pathways by expressing ligands, such as CD80 and PD-L1, which rewire and deactivate T cells within the tumor microenvironment leading to immune escape. ICIs inhibit these interactions, and by reactivation of T lymphocytes, they enable an immune response that targets cancer cells [7].

T cells express the immune checkpoint proteins PD-1 and CTLA-4, which are critical for negative regulation of immune responses. Tumor cells can exploit these pathways by expressing inhibitory ligands that suppress T-cell function and facilitate immune evasion. ICIs are antibodies that target and block such interactions, enabling the immune system to detect and destroy tumor cells. Although this therapeutic strategy has proven to be highly effective, intensive immune activation can also result in a variety of immune-related adverse effects [6].

ICIs that are most used in clinical practice belong to three key groups. These include CTLA-4, PD-1, and PD-L1 target agents. All three classes have shown significant efficacy resulting in increased progression-free survival and overall survival in multiple cancer types.

Therapeutic strategies may include monotherapy or combination therapy. Combination regimens, especially those with simultaneous blockade of both CTLA-4 and PD-1 pathways, have demonstrated significantly improved clinical benefit over monotherapies. For instance, one study showed a 58% higher response rate, and the overall survival lasted 11.5 months longer in patients receiving combination therapy [7].

The targeted immune response against cancer is not without consequences, as blockade of immune checkpoints can result in unwanted immune-mediated damage to healthy tissues, collectively referred to as immune-related adverse events (irAEs). In contrast to the more predictable side effects of conventional cancer therapies, those induced by immune checkpoint inhibitors are varied and can involve several organs [6].

The most common irAEs are dermal, with rash or itching; gastrointestinal, resulting in diarrhea or colitis; or endocrine, leading to thyroid dysfunction, pituitary inflammation, or adrenal insufficiency. Other more serious immune-related toxicity may also be present including pneumonitis, myocarditis, neurological symptoms, myositis, nephritis, and ocular tissue involvement [8].

As summarized in Table 1, CTLA-4 inhibitors such as ipilimumab are more commonly associated with systemic irAEs, while PD-1/PD-L1 blockade may result in a broader, although generally milder, spectrum of organ-specific adverse effects.

### 3.2. Ocular Side Effects of Immune Checkpoint Inhibitors

An analysis of the American Academy of Ophthalmology’s IRIS^®^ Registry revealed that patients receiving ICIs have a markedly increased rate of ophthalmic immune-related adverse events compared with the general population [8].

Ocular side effects during ICI therapy are rare, with approximately 1% of patients affected, most commonly in the form of dry eye disease, conjunctivitis, and keratitis [11]. These ocular side effects are generally mild to moderate in severity and tend to resolve spontaneously or with minimal treatment [10]. Ocular complications that are less frequently observed include corneal perforation, corneal ulcers, pseudomembrane formation, blepharitis, episcleritis, persistent epithelial defects, and rejection of corneal grafts [12].

Complications arising from superinfection of irAEs seem to be strikingly rare as large series of cases would be expected to yield reports by now, and only very rare occurrences are described. Pathogen entry is more likely when immune-mediated inflammation targets epithelial or mucosal barriers, as in the cases of severe keratitis or colitis, while such manipulation of homeostasis is further exacerbated by the initiation of treatment with the immunosuppressive regimens, systemic corticosteroids, or whatever agent is being used to rein in the irAEs. Keratitis, secondary to bacterial infection following ICI-induced uveitis, constitutes another rare case that threatens vision [8,12]. In this case, delayed ophthalmological assessment contributed to the establishment of bacterial keratitis due to nonadherence with follow-up recommendations by the patient.

Dry eye symptoms have been reported in patients treated with Ipilimumab, Nivolumab, Pembrolizumab, Atezolizumab, Avelumab, and Durvalumab. The pathophysiological mechanism is a drug-associated autoimmune impairment of the lacrimal glands [12].

Uveitis, affecting approximately 1% of patients, occurs typically 9 weeks after initiation of ICI therapy [10]. Symptoms may include blurred vision, redness of the conjunctiva, photophobia, and eye pain.

Other forms of uveitis associated with ICIs are birdshot-like uveitis, nonspecific uveitis, choroiditis, and bilateral diffuse melanocytic proliferation and Vogt–Koyanagi–Harada (VKH)-like uveitis [13]. VKH-like uveitis is characterized by exudative retinal detachments. It is hypothesized to depend on the immune cross-reactivity between melanoma antigens and self-antigens on melanocytes, resulting in a T-cell-mediated assault on the choroidal tissues [14].

The precise mechanism inducing to the breakdown of autoimmunity to induce uveitis is unknown; however, there is some evidence that a type II hypersensitivity reaction is involved. For example, in a mouse model of inflammation of the pituitary gland, there was restricted expression of C4 and C3 complement proteins on prolactin- and TSH-secreting target cells that express CTLA-4 [14]. An increase in anti-prolactin and anti-TSH antibodies was detected in patients with hypophysitis after ipilimumab administration [15].

It is hypothesized that anti-CTLA-4 antibodies initially interact with CTLA-4 antigens on pituitary cells. Their Fc regions then bind to C1q, leading to activation of the classical complement pathway. This leads to deposition of C3, C3d, and C4d; generation of the terminal complex; and cell lysis. Antigen presentation then occurs when the damaged cells are ingested by antigen-presenting cells, resulting in the production of serum autoantibodies and further autoimmune attacks toward self-antigens [9]. We did not perform serum or tissue complement assays in this patient; therefore, any role of complement activation remains a hypothesis rather than a demonstrated mechanism.

Recent mechanistic studies have documented that irAEs stem from converging aberrant cytokine production and dysregulated key intracellular signaling pathways [16,17,18,19]. Sustained maintenance of prolonged activation state in both effector T cells and myeloid cells by pro-inflammatory cytokines, such as IL-1β, IL-6, IFN-γ, and TNF-α, constitutes an aberrant pro-inflammatory cytokine milieu for driving autoreactive lymphocyte recruitment into affected tissues including the eye; it also disrupts the blood–ocular barrier, enhances complement activation, and engenders local tissue injury, thereby providing a mechanism for disease recurrence or sustained inflammation. In the ocular case, like our case, these processes may explain the rapid steroid responsiveness once surgical source control was achieved.

irAEs requires integrated approaches embracing multiple omics that can capture interplay among host genetics, functions of immune cells, networks of cytokines, tissue microenvironments, and the microbiome [20]. Unified analysis of genomic, transcriptomic, proteomic, metabolomic, and microbiomics data will allow identifying biomarkers predicting the susceptibility to irAEs, detecting early molecular evidence of toxicity before clinical onset, and informing individualized treatment strategies. In future studies, testing such profiling within ICI trials may allow a pre-treatment risk stratification and provide for real-time monitoring of inflammatory trajectories and precision modulation of immunotherapy to balance anti-tumor efficacy with protection from serious toxicities. In our case, the clear temporal association with PD-1 blockade, exclusion of other infectious, vascular, or immune-mediated causes, and rapid resolution of symptoms after corticosteroids entry lead us to assume that these systemic and ocular manifestations were indeed driven by underlying immune dysregulation.

This class II hypersensitivity model could potentially provide some insight into why ipilimumab, more commonly than other immune checkpoint inhibitors, induces treatment-related toxicity. Ipilimumab is an IgG1 anticlass labeled antibody, which has high affinity for Fc receptors and is the most potent subclass of IgG for antibody-dependent cell-mediated cytotoxicity. In comparison, tremelimumab, a CTLA-4 inhibitor, is also an IgG2 mAb but has less interaction with the Fc receptor system and is a poor activator of the complement system. When compared with the placebo, in clinical trials, tremelimumab seems to have a more favorable IRAEs profile [21].

The retinal involvements with the most well-characterized features at present are the cancer-associated retinopathy (CAR) and the melanoma-associated retinopathy (MAR). Each of these entities is presumed to be triggered by molecular mimicry in which retinal proteins are inappropriately subjected to an immune attack because of their resemblance to tumor antigens. They two retinopathies often have similar symptoms including painless vision loss or blurring, light sensitivity (dysphotopsia), night blindness (nyctalopia), visual distortion (metamorphopsia), and size perception alteration (micropsia), and their onset may occur as soon as 2 weeks after initiation of ICI therapy but up to 2 years since stopping treatment [22].

Ocular irAEs associated with pembrolizumab, such as MAR and CAR, are thought to occur via molecular mimicry, whereby tumor antigens that share structural similarities to retinal proteins trigger a cross-reactive autoimmune response [11,22,23]. This is further substantiated by the detection of anti-retinal antibodies in some patients. Nevertheless, the clinical picture in this case—uveitis complicated by bacterial keratitis and subsequent corneal perforation—is not consistent with one caused by a molecular mimicry-mediated disease. Rather, this situation appears to resemble an immune-mediated ocular inflammation caused by ICI that is worsened by a secondary infection.

These syndromes may be differentiated by cancer type and antibodies associated with each of them: MAR is seen only in patients with melanoma, and CAR is typically found in patients with carcinoma and small cell lung cancer, but also rarely in melanoma, whereas exudative polymorphous vitelliform maculopathy (AEPVM) may be seen with either of the two cancers. The incidence and prevalence of these syndromes in different cancers have not been established [11].

While the net result is mostly undisputed, the role of ICI therapy in the development of these paraneoplastic phenomena remains somewhat elusive. More in detail, MAR is characterized by an immune attack on bipolar cells, CAR to photoreceptors, and acute exudative paraneoplastic vitelliform maculopathy to retinal pigment epithelium cells [23]. Despite targeting different anatomical structures, these three conditions share a common antibody repertoire, and more studies are needed to investigate the processes behind each individual manifestation [24].

Since these ocular disorders may occur even unrelated to ICI treatment, it has been suggested that ICIs could simply unmask an underlying subclinical disease by releasing the brake on immunity. However, autoantibodies are found in only 50–65% of patients, suggesting that different immune mechanisms are involved [25].

A wide spectrum of ocular irAEs has been reported in patients treated with ICIs, with variable presentations, time of onset, and outcomes. Table 2 summarizes key clinical features, implicated agents, prognosis, and suggested management strategies.

### 3.3. Diagnosis

A diagnosis of ICI-induced uveitis requires a definite temporal association with the start of therapy and most of the time requires the exclusion of infectious and other immune-mediated causes. A detailed diagnostic workup is mandatory [26] including searching for autoimmune markers, such as antinuclear antibodies, antineutrophil cytoplasmic antibodies, and rheumatoid factor. Soluble angiotensin-converting enzyme is helpful in the diagnosis of sarcoidosis, which is associated with uveitis in 30–70% of cases.

Single-gene testing for HLA markers associated with a risk of autoimmunity can also be considered.

For instance, spondyloarthropathies share a known association with HLA-B27 and acute anterior uveitis, and HLA-A29 seems to be associated to birdshot chorioretinopathy [28], while HLA-DR4/DRB1 patients are diagnosed more frequently with VKH-like uveitis [27].

irAEs due to ICIs are rare conditions that can present with symptoms, including vision loss, visual field defects, and loss of color vision [13]. Distinguishing between irAEs and a concomitant autoimmune condition is challenging, as the eye and fundus examination are generally not useful.

Electroretinography can aid in the diagnosis of MAR. A characteristic feature in MAR is the markedly reduced or absent b-waves (representing “on” bipolar cell function) with preserved a-waves (representing photoreceptor function) [28].

There are commercially available antibody panels for identifying retinal autoantibodies, but these are not routinely used in a clinical setting [24].

### 3.4. Management

Once ocular toxicity manifests, the decision to continue therapy with ICI therapy should be made on an individual basis.

The management of ICI-induced dry eye disease involves artificial tears (ideally lipid-based to help stabilize the tear film) and topical cyclosporine to decrease T-cell-mediated inflammation. Other more conventional treatment approaches may also be considered [25].

The guidelines of the American Society of Clinical Oncology guidelines on the management of ICI-induced uveitis vary depending on its severity [26]. Anterior uveitis with CTLA-4 inhibitors is generally mild, and in these cases, discontinuation of ICI therapy or topical steroids, cycloplegics, and/or systemic corticosteroids can all be recommended. Nevertheless, in the majority of mild cases corticosteroids will be sufficient with no need to discontinue ICI therapy [22].

Management of AEPVM is less challenging than MAR and CAR. AEPVM is usually reversible with a good prognosis and ICI should be continued because a decreased tumor burden facilitates AEPVM resolution [27].

In contrast, MAR and CAR result in permanent retinal damage with irreversible vision loss as the retina has very low regenerative potential [11].

While clinicians may in specific cases continue therapy, ICI discontinuation is necessary owing to serious, and frequently irreversible, visual impairment. The treatment is based on other immunosuppressive or immunomodulatory approaches.

Unfortunately, these options make patients more vulnerable to opportunistic infections [29].

## 4. Conclusions

The present clinical case illustrates that patients receiving ICIs should be closely monitored, especially when manifesting persistent or atypical symptoms. Chronic refractory diarrhea with severe uveitis and corneal perforation are examples of severe irAEs that appear to respond rapidly to corticosteroid therapy.

The occurrence of irAEs, even of rare ocular manifestations, requires that the decision to continue immunotherapy is appropriately weighted against its toxicity. To prevent long-lasting adverse effects and permanent deficits, a multidisciplinary approach is necessary for the correct diagnosis and timely management.

## Figures and Tables

**Table 1:** Summary of immune checkpoint inhibitors: Targets, mechanisms, and common irAEs.

Checkpoint Pathway	Target Molecule	Representative Drugs	Mechanism of Action	Common irAEs	References
CTLA-4	CTLA-4 on T cells	Ipilimumab, Tremelimumab	Blocks CTLA-4/B7 interaction → enhances T-cell priming	Colitis, dermatitis, hypophysitis	[5,6,9]
PD-1	PD-1 on T, B, and NK cells	Nivolumab, Pembrolizumab	Inhibits PD-1/PD-L1 binding → reverses T-cell exhaustion	Pneumonitis, thyroiditis, hepatitis, uveitis	[5,6,10]
PD-L1	PD-L1 on tumor and immune cells	Atezolizumab, Durvalumab, Avelumab	Prevents PD-L1 from binding PD-1 → preserves T-cell activity	Like PD-1 inhibitors; generally milder	[5,6,8]

**Table 2:** Summary of major ocular immune-related adverse events associated with immune checkpoint inhibitors, including clinical presentation, implicated agents, typical time of onset, prognosis, and management strategies. The table highlights both common and rare events, ranging from dry eye disease to paraneoplastic retinopathies such as MAR and CAR.

Ocular irAE	Clinical Features	Associated ICIs	Time of Onset	Prognosis	Management	References
Dry eye disease	Grittiness, foreign body sensation, blurry vision	All (PD-1, PD-L1, CTLA-4)	Weeks to months	Good	Artificial tears, topical cyclosporine	[12,25]
Uveitis (anterior/posterior)	Red eye, photophobia, eye pain, blurred vision	PD-1 (Nivolumab, Pembrolizumab), CTLA-4	Median: 9 weeks	Variable	Topical/systemic steroids, +/− ICI suspension	[10,13,26]
VKH-like syndrome	Bilateral serous retinal detachments, headache, tinnitus	PD-1, CTLA-4	Weeks to months	Moderate to poor	High-dose corticosteroids, ICI discontinuation	[14]
Corneal ulcer/perforation	Severe pain, photophobia, loss of vision	PD-1 (Pembrolizumab), rare with others	Variable	Often poor (may require evisceration)	Surgical repair, antibiotics, steroids	[12]
Melanoma-associated retinopathy (MAR)	Night blindness, shimmering, central scotomas, preserved a-wave on ERG	PD-1 in melanoma	2 weeks to 2 years post-ICI	Poor (irreversible vision loss)	Immunosuppressants, ICI discontinuation	[22,23]
Cancer-associated retinopathy (CAR)	Painless vision loss, visual field constriction, photopsias	PD-1, rarely CTLA-4	Variable	Poor	Limited response to therapy	[22,24]
AEPVM (vitelliform maculopathy)	Bilateral subretinal fluid, yellowish lesions, mild visual symptoms	PD-1, PD-L1, melanoma or carcinoma	Early to mid-course	Generally good	Often self-resolving; continue ICI	[11,27]

## Data Availability

The data presented in this study are available on request from the corresponding author. The data are not publicly available due to privacy.
